# An algorithm for longitudinal registration of PET/CT images acquired during neoadjuvant chemotherapy in breast cancer: preliminary results

**DOI:** 10.1186/2191-219X-2-62

**Published:** 2012-11-16

**Authors:** Xia Li, Richard G Abramson, Lori R Arlinghaus, Anuradha Bapsi Chakravarthy, Vandana Abramson, Ingrid Mayer, Jaime Farley, Dominique Delbeke, Thomas E Yankeelov

**Affiliations:** 1 Vanderbilt University Institute of Imaging Science, Vanderbilt University, 1161 21st Avenue South, AA-1105 Medical Center North, Nashville, TN 37232-2310, USA; 2 Department of Radiology and Radiological Sciences, Vanderbilt University, 1211 Medical Center Drive, Nashville, TN 37232, USA; 3 Department of Radiation Oncology, Vanderbilt University,, Nashville, TN 37232, USA; 4 Medical Oncology, Vanderbilt University, Nashville, TN 37232, USA; 5 Biomedical Engineering, School of Engineering, Vanderbilt University, Nashville, TN 37235-1826, USA; 6 Department of Physics and Astronomy, Vanderbilt University, Nashville, TN 37235, USA; 7 Cancer Biology, Vanderbilt University, Nashville, TN 37235, USA

**Keywords:** breast cancer, longitudinal registration, FDG-PET/CT, treatment response, metabolic monitoring

## Abstract

**Background:**

By providing estimates of tumor glucose metabolism, ^18^F-fluorodeoxyglucose positron emission tomography (FDG-PET) can potentially characterize the response of breast tumors to treatment. To assess therapy response, serial measurements of FDG-PET parameters (derived from static and/or dynamic images) can be obtained at different time points during the course of treatment. However, most studies track the changes in average parameter values obtained from the whole tumor, thereby discarding all spatial information manifested in tumor heterogeneity. Here, we propose a method whereby serially acquired FDG-PET breast data sets can be spatially co-registered to enable the spatial comparison of parameter maps at the voxel level.

**Methods:**

The goal is to optimally register normal tissues while simultaneously preventing tumor distortion. In order to accomplish this, we constructed a PET support device to enable PET/CT imaging of the breasts of ten patients in the prone position and applied a mutual information-based rigid body registration followed by a non-rigid registration. The non-rigid registration algorithm extended the adaptive bases algorithm (ABA) by incorporating a tumor volume-preserving constraint, which computed the Jacobian determinant over the tumor regions as outlined on the PET/CT images, into the cost function. We tested this approach on ten breast cancer patients undergoing neoadjuvant chemotherapy.

**Results:**

By both qualitative and quantitative evaluation, our constrained algorithm yielded significantly less tumor distortion than the unconstrained algorithm: considering the tumor volume determined from standard uptake value maps, the post-registration median tumor volume changes, and the 25th and 75th quantiles were 3.42% (0%, 13.39%) and 16.93% (9.21%, 49.93%) for the constrained and unconstrained algorithms, respectively (*p* = 0.002), while the bending energy (a measure of the smoothness of the deformation) was 0.0015 (0.0005, 0.012) and 0.017 (0.005, 0.044), respectively (*p* = 0.005).

**Conclusion:**

The results indicate that the constrained ABA algorithm can accurately align prone breast FDG-PET images acquired at different time points while keeping the tumor from being substantially compressed or distorted.

**Trial registration:**

NCT00474604

## Background

^18^F-fluorodeoxyglucose positron emission tomography (FDG-PET) can provide estimates of parameters related to the delivery, retention, and metabolism of glucose, and therefore has been proposed as a method of characterizing the response of tumors to treatment [1-4]. To assess treatment response, semi-quantitative parameters, such as the standard uptake value (SUV) which is derived from static images [5,6], or quantitative parameters which are derived from dynamic images, [7-9], are measured at different time points during the course of treatment. Changes in these values on a region of interest (ROI) basis are then used to assess or predict the response of tumors to therapy [10,11]. In recent years, there has been increasing interest in assessing imaging data at the voxel level, rather than at the ROI level, with the hypothesis that important information on tumor heterogeneity is discarded when an ROI average is performed [12,13]. In order to optimally perform such an analysis during therapy, the data sets acquired at different time points must be accurately co-registered so that similar sections of tissues can be compared. Towards this end, we have introduced a technique that allows for co-registering breast MRI data acquired at different imaging sessions during therapy [14,15]. In this effort, we seek to amend this method to perform longitudinal registration of PET/CT data of the breast in order to enable voxel level analysis of changes observed in FDG-PET images acquired during therapy.

The motivation for developing a method for characterizing changes in FDG-PET scans at the voxel level comes from the fact that probing tumor heterogeneity, as well as changes in that heterogeneity over time, is gaining prominence as a research topic [16]. As MRI studies have shown changes in heterogeneity are predictive of response [13], it is natural to apply a similar approach in studying FDG-PET data. Indeed, as FDG-PET is becoming more accepted in assessing changes in tumor metabolism over time (see, e.g., the most recent version of the Response Evaluation Criteria in Solid Tumors, RECIST [17,18]), the ability to compare the same section of tissue will become increasingly important. While the limited spatial resolution of PET (compared to, e.g., MRI and CT) is a potential barrier for performing such a comparison, it certainly must be addressed to determine if such a promising approach is viable. As has been noted recently [19], volumes of interest established just prior to the initiation of therapy (i.e., baseline images) are not easily transformed to images acquired at later time points, thereby making attempts to study changes in heterogeneity challenging. In this effort, we present one approach to potentially mitigate such difficulties.

There are three main challenges in performing longitudinal registration of breast FDG-PET images: (1) differences in patient positioning between consecutive imaging sessions, (2) changes in tumor shape and volume between imaging sessions, and (3) the relatively low spatial resolution of PET images. To minimize the error introduced by differences in patient positioning, we have designed a support device that allows for PET imaging in the prone position. This allows for the breasts to lay pendant during the scanning session, rather than flat against the chest as in the supine position. It is important to note that we are not the first to introduce this technique; Moy et al. [20] have used prone PET imaging of the breast to facilitate registration of PET data to MRI data, where it has been applied to assist in diagnosis [21,22]. The second problem, addressing the changes in tumor shape and volume that occur due to therapy, requires a registration technique that can maximally align the breast volumes while minimally distorting the tumor, the volume of which must be kept true to what is measured at each time point [14,15]. That is, if *V*(*t*_1_) is the tumor volume at time *t*_1_ (e.g., pre-treatment), and *V*_R_(*t*_1_) is the tumor volume after registration to the post-treatment image, then the registration scheme should minimize the differences between *V*(*t*_1_) and *V*_*R*_(*t*_1_). Similar comments apply for time *t*_2_. To achieve this, we developed and applied a spatially constrained, non-rigid registration method previously used for longitudinal registration of MR images, which incorporates a tumor volume-preserving constraint. The third problem is addressed by applying the algorithm on the computed tomography (CT) images that are acquired during the PET/CT acquisition and applying the resulting transformation to the PET data. We tested our approach on ten patients receiving neoadjuvant chemotherapy for breast cancer, who were scanned at three different time points: pre-therapy, after one cycle of therapy, and at the conclusion of therapy. The PET/CT data at the first two time points were co-registered to the data at the third time point by applying constrained and unconstrained registration algorithms. Both qualitative visual comparisons, as well as a quantitative analysis of the change in the median tumor volume determined from the SUV maps and bending energy obtained from the deformation fields (DF), were employed to evaluate the performance of the registration algorithms.

## Methods

### Patient population

Data were acquired from ten patients with locally advanced breast cancer, who were enrolled in an ongoing phase II clinical trial that is investigating whether the addition of an mTOR inhibitor (RAD001) would have synergistic effects with cisplatin in triple negative breast cancer. The patients provided informed consent, and the study was approved by our Institutional Review Board. Patients with measurable clinical stage II/III triple negative breast cancer are assigned (2:1) to cisplatin 25 mg/m^2^ with and without RAD001 30 mg weekly for 3 weeks, followed by cisplatin + paclitaxel 80 mg/m^2^ with and without RAD001 weekly for 9 weeks until the time of definitive surgery. FDG-PET/CT images of the patients were obtained before (*t*_1_), after one cycle (*t*_2_), and at the completion of neoadjuvant chemotherapy (*t*_3_), yielding a total of 30 data sets (ten patients × three time points). For each patient, we registered the data from *t*_1_ to *t*_3_ and the data from *t*_2_ to *t*_3_, thereby yielding 20 data sets in which to test the registration algorithms.

### Data acquisition

**Figure 1:**
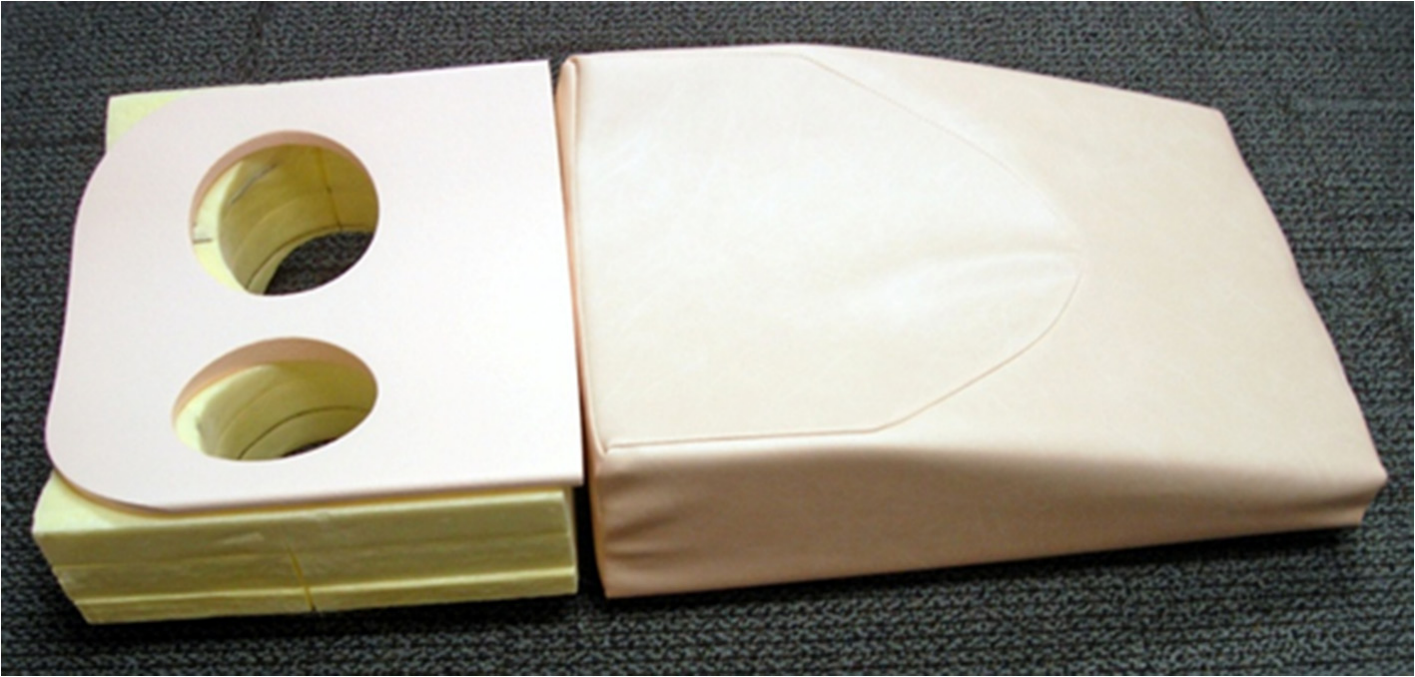
**Our own support for prone breast PET/CT images.** It allows for the breasts to lie pendant during the scanning procedure, therefore greatly enhancing the ability to perform longitudinal registration. Typical breast PET/CT is performed in the supine position which results in less reproducible patient positioning between scan sessions.

Figure 1 displays the specially designed device to enable PET imaging of the breast in the prone position. It is an exact geometric replica of the 4-channel receiver double-breast radiofrequency coil (In vivo Inc., Gainesville, FL, USA) used on our Philips 3T Achieva MR scanner (Philips Healthcare, Best, The Netherlands). A separate, ongoing study is examining the ability to facilitate co-registration of FDG-PET data with MRI data. The rigidity of the device is provided by lightweight, rigid polystyrene foam insulation (part # 9255K3; McMaster, Atlanta, GA, USA), which was purchased in bulk and machined to match the dimensions of the breast coil. To provide patient comfort, a set of the padding used to support patients for the MRI coil was purchased and affixed to the support (please see Figure 1). Once constructed, the support device was assessed for CT attenuation by certified PET/CT technologists. For a similar approach and design, please see the work by Moy et al. [20-22].

PET/CT data were acquired with a GE Discovery STE scanner (GE Healthcare, Waukesha, WI, USA). A low-mAs CT scan was acquired for attenuation correction of the emission data. The acquisition parameters for the transmission CT scan were the following: the tube current was 80 mAs for a 70-kg patient and scaled accordingly for all patients, the tube voltage was 120 KVp, and the pitch was 1.675/1. The activity of FDG administered was approximately 370 MBq (10 mCi) for a 70-kg patient and scaled according to weight. FDG was administered intravenously *via* an antecubital vein contra-lateral to the affected breast. After 60 min, emission data was collected in 3D mode for 2 min per bed position. The emission scan was first collected in the prone position over the breast only, and then in the supine position from the skull to mid-femurs. Standard-of-care supine images and research prone images were acquired at times *t*_1_ and *t*_3_, while only the prone images were acquired at *t*_2_.

### Image registration methods

While details of our constrained, non-rigid registration algorithm for alignment of breast images acquired during therapy technique can be found elsewhere [14,15], we briefly describe the salient features and enhancements required to apply the approach to PET/CT data. A rigid body registration algorithm [23] is first used to align the breast CT images at *t*_1_ and *t*_2_ to the target images at *t*_3_. This algorithm searches the optimal rotation and translation parameters through maximizing the normalized mutual information (NMI, Equation 1):

(1) $$NMI=\frac{H\left(A\right)+H\left(B\right)}{H\left(A,B\right)}\text{,}$$

where *H*(A) and *H*(B) are the marginal entropy of images A and B, and *H*(A,B) is the joint entropy. A non-rigid registration method [14] is then applied to the longitudinal breast CT images between the first two time points and the third. This method relies on the adaptive bases algorithm (ABA) [24]. The ABA algorithm employs NMI as the similarity measure, and the deformation field is modeled by a linear combination of radial basis functions [25]. Through searching the optimal coefficients of radial basis functions, the NMI can be maximized, and the deformation field that registers the two images can be obtained. The ABA algorithm is applied using a hierarchical multi-resolution strategy, in which the original PET/CT images are down-sampled into a number of low-resolution images, and the registration starts at a low resolution level, with few basis functions, and ends at the highest resolution level.

For the present application, we extend the ABA algorithm by incorporating an additional term (i.e., the constraint) designed to preserve the tumor volume during the registration process in the cost function. This term is computed as the Jacobian determinant over the tumor regions as outlined on the PET/CT (described below) images; hence, the cost function is composed of two terms: the negative NMI term and the tumor volume constraint term:

(2) $$f_{\cos t}=-NMI+\alpha \int_{T}\left|\log\left(J_{T}\left(x\right)\right)\right|dx\text{,}$$

where *J*_T_(*x*) is the Jacobian determinant on the tumor area, and *α* is the parameter to control the weight of this constraint term. The value of *α* can be adjusted from 0 to 1 based on individual datasets; the smaller the weight, the less the tumor volume is constrained (please see the ‘Discussion’ section for more comments on this technical point). For the patient data sets in this study, a weight of 0.1 was unable to constrain the tumor volume for some patients, while a weight of 0.5 was too strong to allow the reorientation of the tumor in other patients; thus, a weight of 0.3 was (empirically) selected for all 20 pairs of image sets. Through minimizing the cost function, the algorithm optimally registers the normal tissues while simultaneously minimizing tumor distortion.

To perform the constrained registration, the tumor volume must first be segmented. We note that determination of the tumor volume in PET images by thresholding is still an area of active discussion. For example, Drever et al. [26] have shown that the optimal threshold depends mainly on lesion size and contrast, and that a threshold of 40% can be reasonable for larger lesions. However, Biehl et al. [27] showed that the PET and CT gross tumor volumes were approximately equal using a threshold of 40% for tumors less than 3 cm. As an in-depth analysis of the determination of the threshold was outside the scope of the study, we simply segmented the tumor volume using a threshold of 40% of the maximum SUV uptake, a common method to delineate tumor volumes on SUV maps of FDG [28-30]. More specifically, the SUV maps were calculated for each patient and loaded into in-house software (based on MATLAB, Natick, MA, USA). An experienced radiologist drew tumor ROIs large enough to ensure that all possible tumor voxels were included. These manually drawn ROIs may also include some healthy tissue voxels. The ROIs for each slice were then combined to generate 3D masks. The final tumor volume is detected by finding all voxels with SUV larger than 40% of the maximum SUV uptake in the 3D masks. These are the tumor voxels, the Jacobian determinant of which is minimized during the application of the constrained algorithm in order to maximally preserve the tumor volume during the registration process. It is also important to note that our previous studies [15] have shown that the selection of the tumor volume (i.e., the volume of tissue that is to be constrained during the registration process) does not significantly affect the performance of the method.

**Figure 2:**
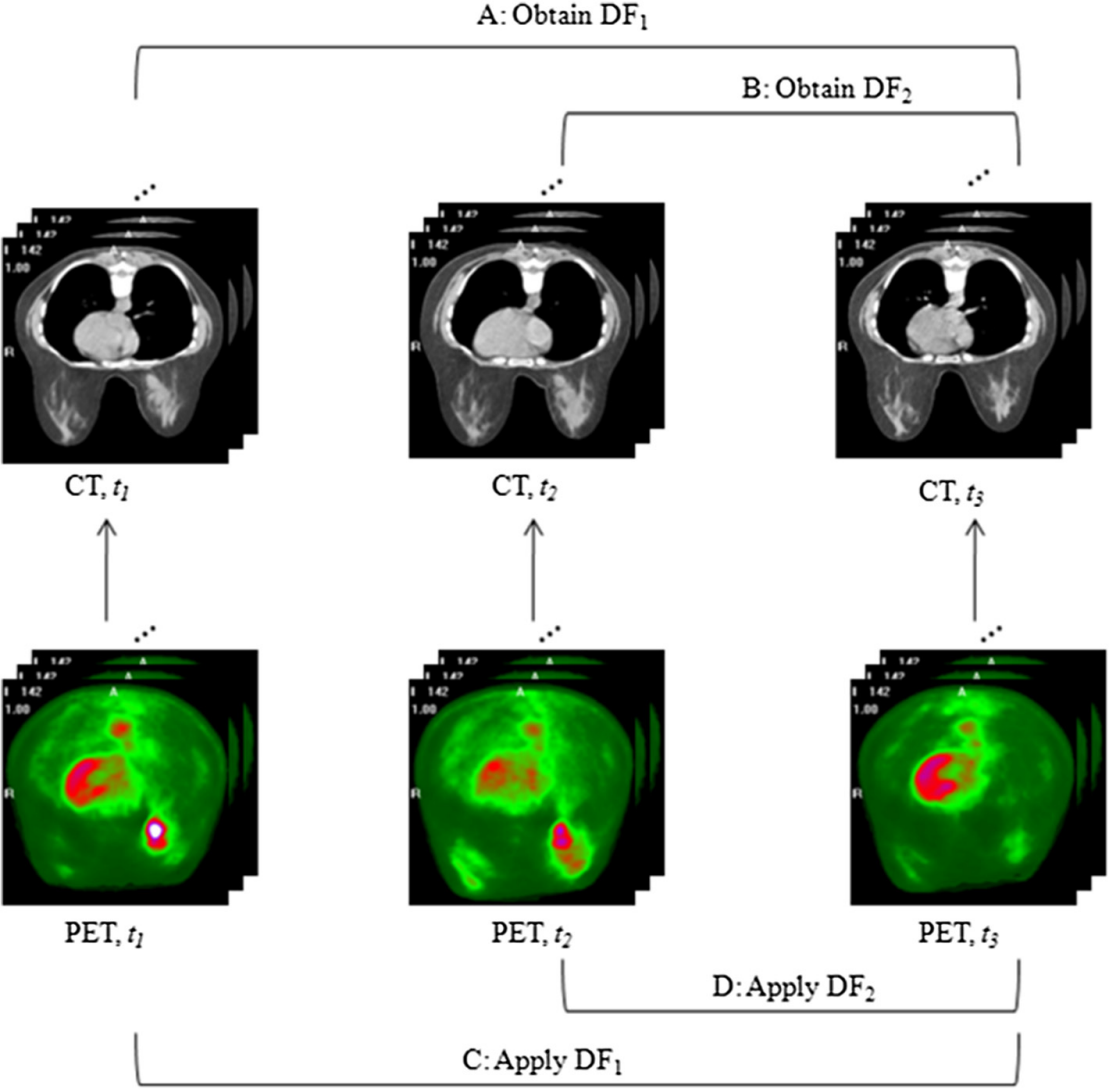
**The scheme for applying the algorithm to register the PET/CT.** The CT data obtained during *t*_1_ and *t*_2_ are aligned via the proposed registration algorithm (steps **A** and **B**) to the CT images acquired at *t*_3_. The resulting DF are then applied to the corresponding PET images to yield co-registered longitudinal PET data (steps **C** and **D**).

Figure 2 shows the scheme for applying the algorithm to register the PET/CT images acquired at the three time points throughout therapy. The CT data at *t*_1_ and *t*_2_ were registered to the CT data at *t*_3_ (though, in principle, any time point could be selected as the ‘target’ to which the other time points are aligned), yielding the deformation fields (steps A and B in the figure). The deformation fields were then applied to the corresponding PET images obtained at *t*_1_ and *t*_2_ (steps C and D), respectively, so that all data are aligned to the *t*_3_ images at the end of the process.

### Validation approach

The validity of the constrained and unconstrained algorithms was tested both qualitatively and quantitatively. Qualitative assessment involved visual comparisons of the PET/CT images and deformation fields before and after registration using the constrained and unconstrained algorithms. Quantitative assessment was performed by calculating (1) the changes in the SUV tumor volumes after registration and (2) the bending energy. The bending energy, which describes the smoothness of the deformation fields [15], is calculated as follows:

(3) $$\begin{array}{l} E_{\text{bending}}=\frac{1}{V}∭({\left(\partial^{2}T/\partial x^{2}\right)}^{2}+{\left(\partial^{2}T/\partial y^{2}\right)}^{2}\\ \;+{\left(\partial^{2}T/\partial z^{2}\right)}^{2}+2{\left(\partial^{2}T/\partial x\partial y\right)}^{2}\\ \;+2{\left(\partial^{2}T/\partial x\partial z\right)}^{2}+2{\left(\partial^{2}T/\partial y\partial z\right)}^{2})dxdydz\text{,} \end{array}$$

where *T* is the transformation, and *V* is the tumor volume.

The percentage change of tumor volumes is used to measure the degree the constrained algorithm preserves tumor volume or the extent the unconstrained algorithm distorts the tumor. When looking at the longitudinal data during therapy response, one of the primary goals is to predict which patient will achieve a positive response. Any change in tumor size should be due to changes in biology and not to changes in patient positioning or errors in the registration scheme. Thus, we want to make sure that changes in the tumor volume are absolutely minimized during registration. Given our earlier result on applying this technique to MRI data, we hypothesized that the tumor volume will be similar before and after applying the constrained ABA registration algorithm (i.e., Equation 2), while the tumor volume will be changed substantially using the unconstrained ABA registration algorithm (i.e., Equation 2 without the second term on the right hand side) because the unconstrained algorithm tends to compress or stretch the tumor at early stages of treatment (i.e., *t*_1_ and *t*_2_) to match the post-treatment (i.e., *t*_3_) tumor size and shape. We also hypothesized that compared with the unconstrained ABA approach, the constrained ABA algorithm will lead to a smoother transformation, and therefore a smaller bending energy.

### Statistical analysis

We used the Lilliefors test to determine if the change of the tumor volumes and the bending energy came from a normal distribution. The test showed that the data did not come from a normal family (*p* < 0.05 for the bending energy obtained by both the constrained and unconstrained algorithms and the tumor volume change obtained by the unconstrained algorithm). Hence, instead of using the mean and standard deviation, the median and the lower and upper quantiles (the 25th and 75th quantiles) were reported for the change of the tumor volumes and the bending energy. The non-parametric Wilcoxon signed rank test was then applied to determine if the results obtained by the constrained and unconstrained ABA algorithms were significantly different.

## Results

### Qualitative assessment of registration algorithms

**Figure 3:**
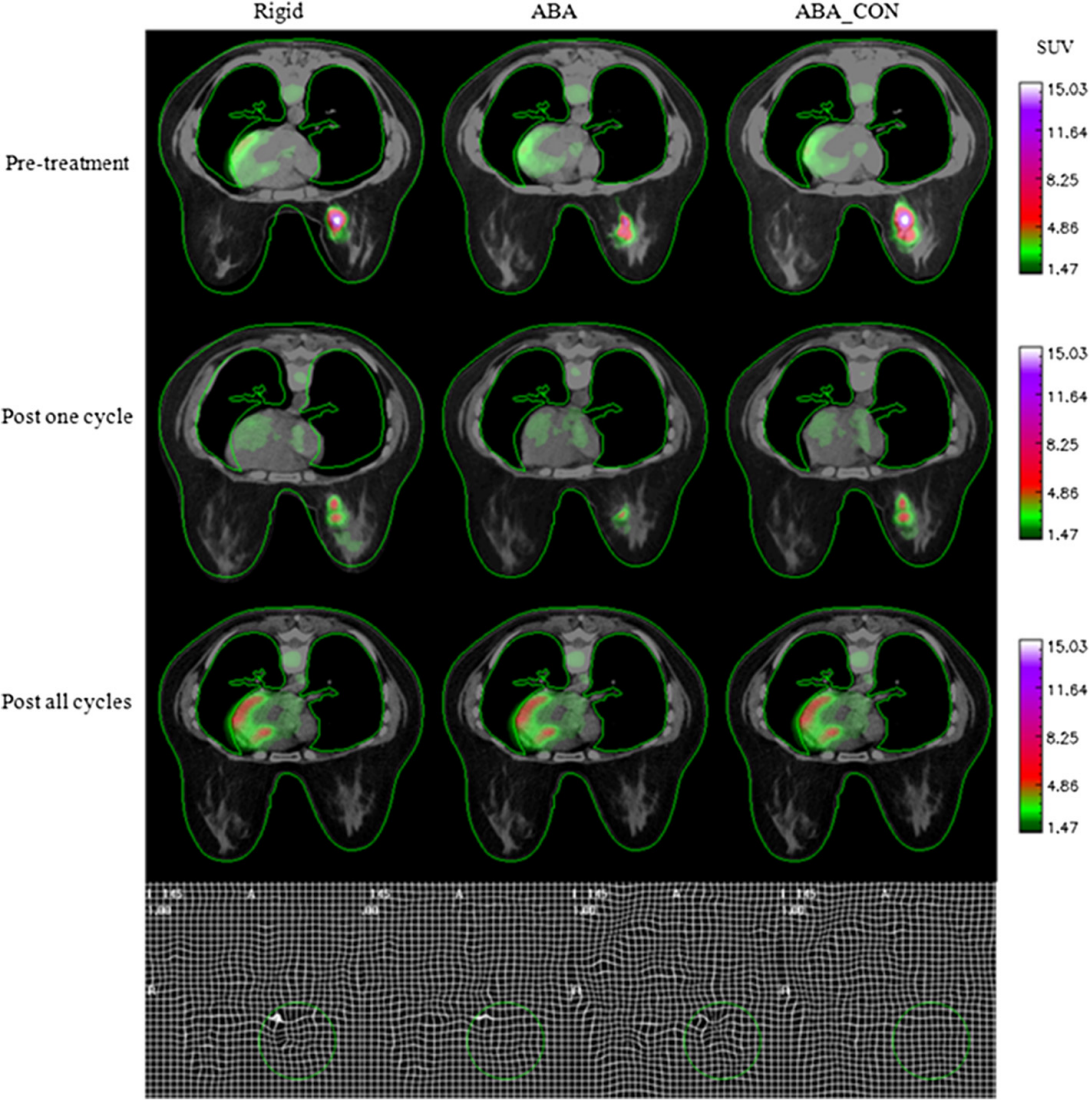
**A representative patient displaying the results of the three registration algorithms.** The first three rows correspond to the three time points, and the three columns show the results after rigid body registration, after unconstrained ABA registration (ABA), and with constrained ABA registration (ABA_CON), respectively. In the fourth row, the first panel displays the deformation field generated by the ABA when the images at *t*_1_ are registered to the images at *t*_3_, while the second panel shows the result using the ABA_CON; the third and fourth panels display similar data when the images at *t*_2_ are registered to the images at *t*_3_, respectively. The green circle shows the tumor location. The contour of the CT image at *t*_3_ is drawn and then copied to other images to facilitate the comparison.

**Figure 4:**
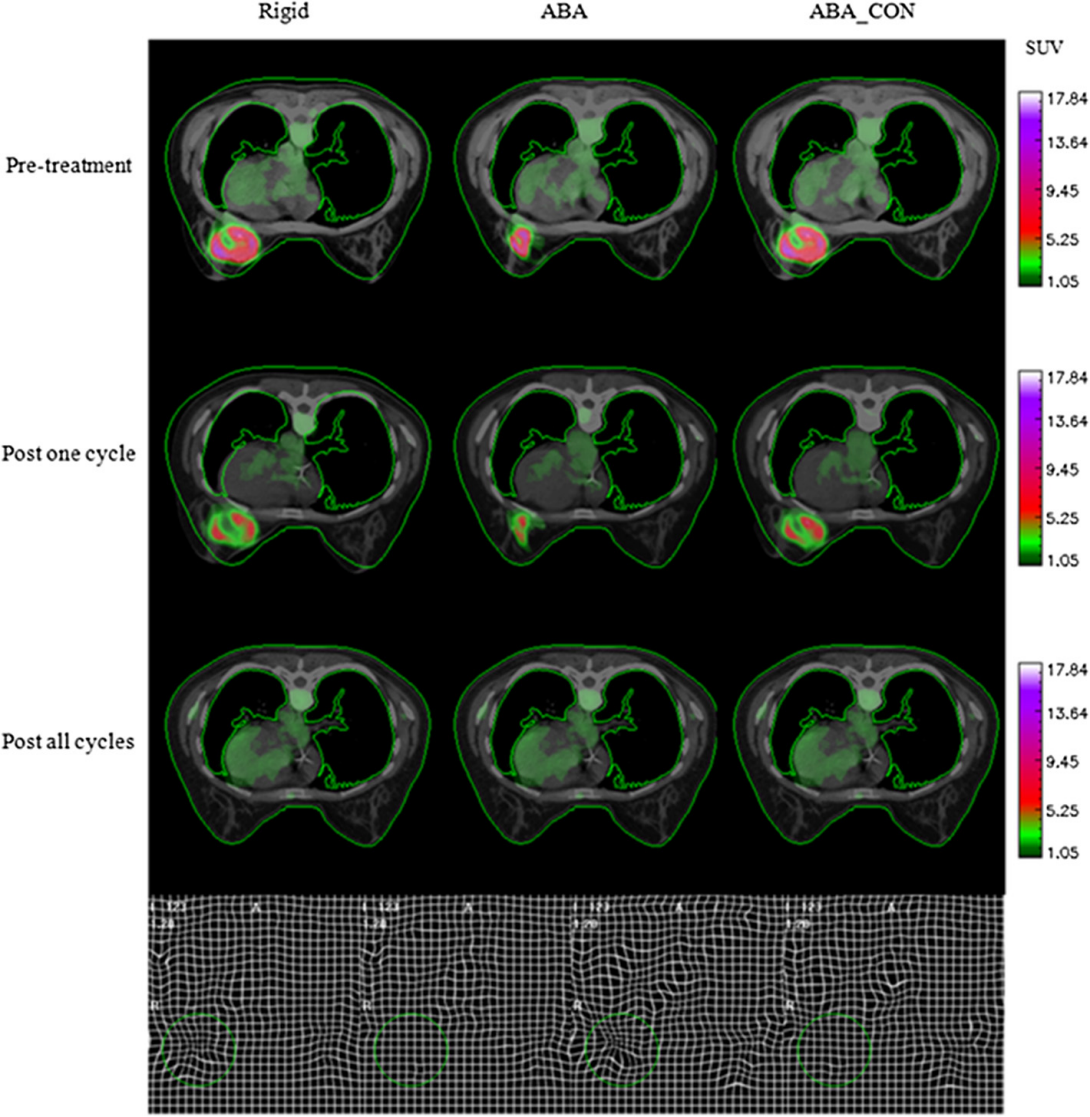
**Results of the three registration algorithms for another patient with similar setup with Figure**3.

In general, the rigid body registration algorithm provided imperfect alignment of breast tissue. The unconstrained ABA provided improved breast tissue registration, but at the expense of dramatically shrinking the tumor to match the tumor shape, whereas the constrained ABA achieved a (qualitatively) satisfactory alignment in the normal tissues with only minimal distortion of the tumor. Figures 3 and 4 display representative registration results for two patients who were each diagnosed with an invasive ductal carcinoma. In each panel, the standardized uptake values are superimposed on the anatomical CT images. The first column of each figure shows three axial slices obtained by aligning the PET/CT images at *t*_1_ (row 1) and *t*_2_ (row 2) to the PET/CT data acquired at *t*_3_ (row 3) *via* a rigid body registration. The second and third columns show the same slices registered by the unconstrained and constrained ABA algorithms, respectively. The contours of the CT images at *t*_3_ were drawn and then copied to other images to facilitate the comparison. In the fourth row, the first panel displays the deformation field generated by the unconstrained algorithm when the images at *t*_1_ were registered to the images at *t*_3_, while the second panel shows the result using the constrained algorithm; the third and fourth panels display similar data when the images at *t*_2_ were registered to the images at *t*_3_, respectively. The first column (left-most) shows that the rigid body registration provided a general alignment of breast tissues imaged at the three time points but was not perfect. Column 2 shows that the unconstrained registration provides an accurate registration of the breast tissues, but only at the expense of dramatically shrinking the tumor observed at times *t*_1_ and *t*_2_ to match the tumor shape at *t*_3_. The constrained algorithm (column 3) achieved a high degree of accuracy in the normal tissues with only minimal distortion of the tumor. Figure 4 displays similar panels, and the results also indicate that the constrained algorithm protects the tumor from large distortion. Column 3 in Figure 4 also shows a slight mismatch in the edge of the diseased breast, since the tumor is large and the constrained term dominates the registration algorithm over the tumor area, leading to the preservation of the tumor volume but to the small mismatch of surrounding tissues. Also note that even though there is no visible tumor in the PET image at *t*_3_, the constrained algorithm still successfully preserves the overall volume and shape of the tumor at *t*_1_ and *t*_2_.

**Figure 5:**
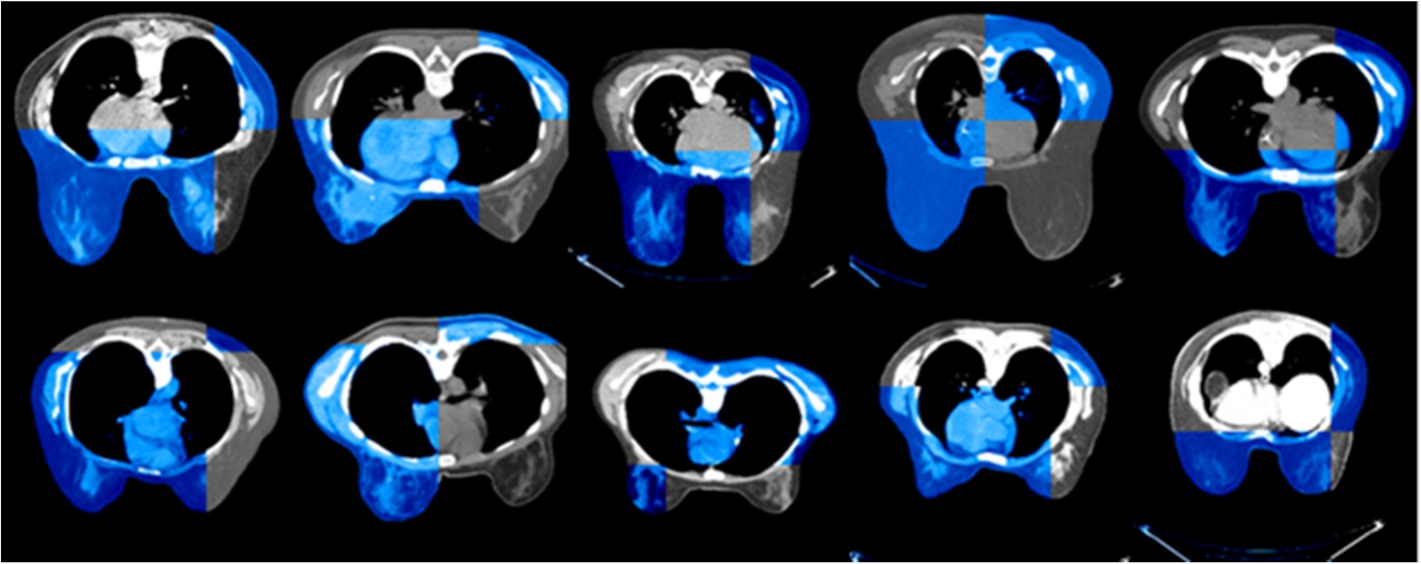
**Ten examples from ten patients illustrating the matching accuracy of the registration algorithm.** Axial CT slices obtained at *t*_1_ or *t*_2_ (colored in blue) are overlaid on the corresponding images obtained at *t*_3_ (gray) in a checkerboard pattern to facilitate assessments of the registration performance. Note that the structural boundaries between blue and gray images are connected accurately, indicating a good performance of the registration algorithm.

Figure 5 shows ten example images from the ten patients in which the CT images obtained at *t*_1_ or *t*_2_ (colored in blue) are overlaid on the images at *t*_3_ (gray) in a checkerboard pattern to facilitate assessments of the registration performance. Note that the structural boundaries between blue and gray images are connected accurately, indicating the accuracy of the registration algorithm.

### Quantitative assessment of registration algorithms

Returning to Figure 3 (also refer to patient #1 in Table 1), the changes in tumor volume from *t*_1_ to *t*_3_ and from *t*_2_ to *t*_3_ obtained by the constrained ABA algorithm are 0.0% and 7.59%, respectively, while application of the unconstrained ABA algorithm results in tumor volume changes of 63.21% and 78.99%, respectively. The bending energies are 0.002 and 0.0, respectively, for the constrained algorithm, while the unconstrained algorithm results in the bending energies of 0.052 and 0.036, respectively. Similarly, for Figure 4 (also refer to patient #2 in Table 1), the changes in tumor volume from *t*_1_ to *t*_3_ and from *t*_2_ to *t*_3_ obtained by the constrained ABA algorithm are 9.28% and 0.09%, and the bending energies are 0.0 and 0.001, respectively, while the unconstrained algorithm results in tumor volume changes of 63.13% and 61.07%, respectively, and the bending energies of 0.079 and 0.051, respectively. Table 1 lists the bending energies and volume changes for ten patients. For each patient, the data at *t*_1_ was registered to *t*_3_, and the data at *t*_2_ was registered to *t*_3_, yielding a total of 20 datasets. The median and lower and upper quantiles of tumor volume changes are 3.42% (0%, 13.39%) and 16.93% (9.21%, 49.93%) (*p* = 0.002) for the constrained and unconstrained ABA algorithm, respectively, while the median bending energy is 0.0015 (0.0005, 0.012) and 0.017 (0.005, 0.044), respectively (*p* = 0.005). The results indicate that the constrained approach is significantly better than the unconstrained approach in preserving the tumor volume during longitudinal registration.

**Table 1:** The bending energy and change of tumor volumes are calculated using the constrained (ABA_CON) and unconstrained ABA algorithms (ABA), respectively

**Patient number**	**Dataset**	**Bending energy**		**Change of tumor volumes**	
		**ABA**	**ABA_CON**	**ABA (%)**	**ABA_CON (%)**
1	*t*_1_ → *t*_3_	0.052	0.002	63.21	0.00
	*t*_2_ → *t*_3_	0.036	0.000	78.99	7.59
2	*t*_1_ → *t*_3_	0.079	0.000	63.13	9.28
	*t*_2_ → *t*_3_	0.051	0.001	61.07	0.09
3	*t*_1_ → *t*_3_	0.021	0.000	51.01	0.00
	*t*_2_ → *t*_3_	0.036	0.000	48.84	0.48
4	*t*_1_ → *t*_3_	0.057	0.001	16.38	1.43
	*t*_2_ → *t*_3_	0.004	0.001	−6.95	−6.66
5	*t*_1_ → *t*_3_	0.011	0.007	40.15	36.50
	*t*_2_ → *t*_3_	0.012	0.009	−1.30	5.41
6	*t*_1_ → *t*_3_	0.051	0.035	17.27	−19.97
	*t*_2_ → *t*_3_	0.020	0.024	9.89	9.16
7	*t*_1_ → *t*_3_	0.019	0.020	33.33	32.85
	*t*_2_ → *t*_3_	0.006	0.005	12.34	19.23
8	*t*_1_ → *t*_3_	0.015	0.015	16.29	12.45
	*t*_2_ → *t*_3_	0.012	0.020	4.96	0.39
9	*t*_1_ → *t*_3_	0.004	0.003	−5.62	−6.80
	*t*_2_ → *t*_3_	0.001	0.001	16.58	14.32
10	*t*_1_ → *t*_3_	0.001	0.000	26.55	14.55
	*t*_2_ → *t*_3_	0.002	0.001	8.52	−4.92
Median		0.017	0.0015	16.93	3.42
*p* values		0.005		0.002	

The Wilcoxon signed rank test was applied to the data to determine if the results are significantly different (*p* values are listed in the final row). The results demonstrate that the constrained ABA causes smoother deformation fields and preserves tumor volumes significantly.

## Discussion

In previous studies [14,15], we presented a tumor volume-preserving registration algorithm for breast MR images acquired at different time points during therapy, validating this algorithm quantitatively on both simulated and experimental breast MR data. The tumor volume changes obtained for MR images were 4.8% and 46.9% for the constrained and unconstrained algorithms, respectively, and the bending energies were 0.0058 and 0.15, respectively [15]. The accurate performance of this algorithm on MR data makes it a promising technique to apply on longitudinally acquired breast data available from other modalities. In this effort, we have applied the constrained, non-rigid registration algorithm to sequential breast PET/CT images acquired during neoadjuvant chemotherapy. Visual assessment (Figures 3, 4, and 5) demonstrates that the algorithm leads to accurate registration results. Quantitative assessment (Table 1) also shows that the algorithm yields smoother deformation fields and significantly less tumor volume change compared with the unconstrained, non-rigid registration algorithm.

One limitation of this study is that the registration algorithm may not correct the change in the whole breast volume over time, e.g., due to hormonal fluctuations over the menstrual cycle [31]. In the current technique, the constrained term is applied to the tumor ROI to preserve the tumor volumes, and NMI is used to perform the alignment. Therefore, this technique may not be able to handle changes in overall breast volume.

We note that there are some negative volume changes in Table 1. This may have occurred since the difference of patient positioning in particular cases could be large, resulting in large deformation during registration between two time points. Under some circumstances, the registration algorithm must stretch the healthy tissue in the source image to match with the target image. When the mutual information term in the cost function is substantially greater than the constraint term, both the healthy tissue and the tumor area will be stretched during registration, and this could result in tumor expansion. We also note that for some patients (e.g., patients #5 and 7 in Table 1), the results obtained by both the constrained and unconstrained algorithms were very similar. One possible reason is that for all 20 data sets, we use similar registration parameter settings (e.g., the number of radial basis functions and the total resolution levels); in particular, we fixed the weight of the constraint term, α, to a value of 0.3. However, as the tumor shape, volume, and normal tissue structures are substantially different between patients, it is highly likely that empirical adjustment of those parameters for different patients would improve the performance of the constrained. One of the future goals is to automate the optimal selection of these parameters. Another future direction is to use MR images to further verify the performance of the constrained algorithm on PET/CT images. As mentioned above, MR images have a higher spatial resolution (than PET) and could be used to examine if larger voxel size or partial volume effect causes the registration inaccuracy in PET/CT data, though applying the transformation obtained from PET/CT data to MR images.

Although currently, most studies still focus on the assessment of physiological parameters at the ROI level, investigators have realized the importance of tumor heterogeneity contained in parametric maps. Maday et al. [12] employed a deformable registration and texture analysis on breast magnetic resonance images to assess the response of breast cancer to therapy and concluded that their approach improved the ability to predict response. Padhani et al. [13] also reported that the change in the range of tumor perfusion and permeability estimated by MRI was able to predict breast cancer response to treatment, indicating that intra-tumoral heterogeneity is important. O'Connor et al. [32] also showed that DCE-MRI biomarkers of tumor heterogeneity may predict the shrinkage of colorectal cancer liver metastasis. Li et al. [33] applied longitudinal registration to DCE-MRI data and performed a voxel-by-voxel analysis. Their preliminary results indicated that the voxel-based analysis may improve the ability of DCE-MRI parameters to separate complete responders from non-responders after one cycle of chemotherapy. Hence, longitudinal registration keeps the information of tumor heterogeneity and spatial distribution of the physiological parameters, thereby enabling spatial comparison at the voxel level. It is a reasonable hypothesis that registration of PET/CT breast images obtained during therapy may allow for an improvement in assessing and/or predicting treatment response. Hence, future efforts will apply the algorithm to parametric maps (e.g., the standard uptake value) to investigate if assessing the changes at the voxel level can improve, over region-of-interest-based measurements, the ability of FDG-PET to predict the response of breast tumors to neoadjuvant chemotherapy. In particular, it will be important to determine if the constrained algorithm can limit registration errors to such a level that they would be acceptable for clinical evaluation based on voxel (or, at least, local) uptake analysis. As our data set grows, this will be a primary goal to address. Such an analysis has been applied in MRI longitudinal studies of treatment response in cancer [33-35], and the methods presented in this contribution enable such an approach for PET data.

## Conclusion

This study indicates that the constrained registration method can accurately align prone breast FDG-PET images acquired at different time points while keeping the tumor from being substantially compressed or distorted. Future studies, in a larger cohort of patients, will test whether the approach can enhance the ability to predict eventual response, thereby by providing an important (clinical) validation.

## Competing interests

The authors declare that they have no competing interests.

## Authors’ contributions

XL contributed to the study design, performed data analysis, and is the primary author of the manuscript. RGA contributed to the study design, performed data interpretation, and contributed substantially to the writing of the manuscript. LRA contributed to the study design and collected all data in the manuscript. ABC, VA, and IM contributed to the study design and data interpretation, and recruited appropriate patients to the study. JF contributed to the study design and assisted LRA in all aspects of data collection. DD contributed to the study design, provided guidance on data analysis, and contributed to the writing of the manuscript. TEY was the primary investigator responsible for the experimental design, intellectual content, supervision of data collection and analysis, and writing/editing the manuscript. All authors reviewed and provided feedback and insights for further research. All authors read and approved the final manuscript.

## Authors’ information

XL and LRA are imaging research scientists. RGA is an assistant professor. ABC is an associate professor and a radiation oncologist. VA is an assistant professor of medicine and a medical oncologist. IM is also an assistant professor of medicine and a medical oncologist. She is the clinical director of the Breast Cancer Program. JF is a licensed practical nurse. DD is a professor of the Radiology and Radiological Sciences Department and the director of the Nuclear Medicine and Positron Emission Tomography. TEY is an associate professor of the Radiology and Radiological Sciences Department, Biomedical Engineering, Physics and Astronomy, and Cancer Biology, and the director of Cancer Imaging Research.
